# Density Functional Theory-Based Indicators to Estimate the Corrosion Potentials of Zinc Alloys in Chlorine-, Oxidizing-, and Sulfur-Harsh Environments

**DOI:** 10.3390/molecules29163790

**Published:** 2024-08-10

**Authors:** Azamat Mukhametov, Insaf Samikov, Elena A. Korznikova, Andrey A. Kistanov

**Affiliations:** 1 The Laboratory of Metals and Alloys Under Extreme Impacts, Ufa University of Science and Technology, 450076 Ufa, Russiaelena.a.korznikova@gmail.com (E.A.K.); 2 Polytechnic Institute (Branch) in Mirny, North-Eastern Federal University, 678170 Mirny, Russia

**Keywords:** dataset, adsorption energy, work function, surface, single-atom adsorption

## Abstract

Nowadays, biodegradable metals and alloys, as well as their corrosion behavior, are of particular interest. The corrosion process of metals and alloys under various harsh conditions can be studied via the investigation of corrosion atom adsorption on metal surfaces. This can be performed using density functional theory-based simulations. Importantly, comprehensive analytical data obtained in simulations including parameters such as adsorption energy, the amount of charge transferred, atomic coordinates, etc., can be utilized in machine learning models to predict corrosion behavior, adsorption ability, catalytic activity, etc., of metals and alloys. In this work, data on the corrosion indicators of Zn surfaces in Cl-, S-, and O-rich harsh environments are collected. A dataset containing adsorption height, adsorption energy, partial density of states, work function values, and electronic charges of individual atoms is presented. In addition, based on these corrosion descriptors, it is found that a Cl-rich environment is less harmful for different Zn surfaces compared to an O-rich environment, and more harmful compared to a S-rich environment.

## 1. Introduction

Surface adsorption is one of the fundamental processes in many fields, including catalysis, the environment, energy, and medicine. In medicine, biodegradable metals and alloys and their corrosion behavior are of particular interest. In turn, the corrosion process of metals and alloys is associated with the adsorption of corrosive atoms to their surfaces. The corrosion process of biodegradable metals and alloys can be studied in an immersion test. In that case, biodegradable metals and alloys are placed in special solutions, such as Hanks’ solution, compositionally similar to blood plasma. These solutions are rich in various minerals, such as sodium, calcium, magnesium carbonates, and bicarbonates [1]. In addition, it is also crucial to understand the corrosion behavior of biodegradable metals and alloys in sulfur- and oxygen-enriched atmospheres [2,3,4]. Nowadays, various computational approaches have become trustable and utilize comparably fast and cheap tools to assess the corrosion behavior of biodegradable metals and alloys [5,6,7,8]. For instance, using density functional theory (DFT)-based modeling, it has been shown that in α-Al_2_O_3_(0001), the insertion of a Cl atom in an aluminum vacancy is an endothermic process and the activation energy for the Cl ingress exceeds 2 eV, while the insertion a Cl atom in an oxygen vacancy is an exothermic process [9]. DFT-based simulations have uncovered the fundamental mechanism of the interaction between pure and doped TiO_2_ with S and O species [10]. Another theoretical investigation of anticorrosion properties of doped Ni-based alloys in Br-rich and O-rich environments can be enhanced via adsorption inhibition [11]. Pure metal surfaces have also been actively studied in terms of corrosion behaviors in different corrosion mediums. Experimental scanning tunneling microscope results combined with DFT calculations have revealed a dynamic process of chlorine adsorption on the Au surface, where Au atoms form a complex superlattice of a Au–Cl surface compound [12]. In the framework of DFT, the coverage of the Fe(100) surface by Cl atoms has been studied [13]. Adsorption energies and buckling distances have been calculated for various coverages as a percentage. Notably, a limited number of works are available on corrosive atom adsorption on Zn surfaces. The DFT results have shown that the S species can chemically interact with Zn atoms at the smithsonite surface and repel water molecules from it [14]. In this study [15], among 22 considered single atoms, Al, Ag, Cd, In, Sn, Au, Hg, Tl, and Bi atoms have been sorted out as doping atoms that possess a weak adsorption energy and low diffusion activation energy on the Zn surface.

Meanwhile, comprehensive analytical data, such as adsorption energy, the amount of charge transferred, atomic coordinates (bond lengths, bond angles, and interatomic distances), etc., can be used in machine learning models as indicators to predict corrosion behavior, adsorption ability, catalytic activity, etc., of materials [16]. For example, the adsorption characteristics of graphene modified using single-atom adsorption has been investigated via DFT-based methods [17]. Various adsorption features, such as atomic and electronic structures, magnetic properties, and adsorption energies of single-atom-modified graphene have been collected and analyzed. These descriptors can be used in machine learning models for the development and design of graphene-based single-atom catalysts. In the work [18], the adsorption of single atoms on the graphene surface of a graphene/aluminum composite was studied using DFT methods supplied with a machine learning approach. A dataset was created containing basic information on atoms, such as atomic radius, ionic radius, etc., as well as adsorption energy and interatomic distances of about thirty atoms at the graphene surface. As a result, it has been shown that single atoms of individual elements, such as Zr, Ti, Sc, and Si can affect the reaction in the interfacial region of graphene and aluminum. Furthermore, the hydrogen storage capacity of MXene materials was tested based on the machine learning models using hydrogen adsorption energy as a main descriptor [19]. Accordingly, several bilayer MXenes with excellent hydrogen adhesion and storage capacities have been designed. Another recent study [20] has introduced the hierarchically interacting particle neural network to predict the energies of molecules based on their physical principles available in the QM9 dataset. Such a method can also work with the data obtained from ab initio molecular dynamics calculations; thus, a dataset can be created based on a specific task. A similar model based on the hierarchically interacting particle neural network has later been used to predict single-atom adsorption on metal and bimetal surfaces [21]. Adsorption energy, adsorption height, and buckling of the surface has been predicted for H, N, and O atoms adsorbed on clean FCC metal surfaces. Some advanced machine learning-based models can also predict the dissociative adsorption energy of single molecules to metal nanoparticles [22] and surfaces [23]. Therefore, the collection of data that will facilitate the development of an adsorption model is a long-term goal in surface and interface science.

This work aims to address two scientific challenges: assessing the corrosion behavior of Zn surfaces in the Cl-, O-, and S-rich harsh environments, and collecting data on the corrosion indicators of Zn surfaces in Cl-, S-, and O-rich harsh environments. A dataset containing the adsorption height *d*, adsorption energy *E*_ads_, partial density of states (PDOS), work function (WF) values, and electronic charges of individual atoms for the single Cl, O, and S atoms on the Zn(111), Zn(110), and Zn(100) surfaces was created. Consequently, based on the data obtained, the corrosion ability of Zn surfaces was evaluated in relation to their tendency to adsorb corrosive Cl, O, and S atoms.

## 2. Results

**Figure 1:**
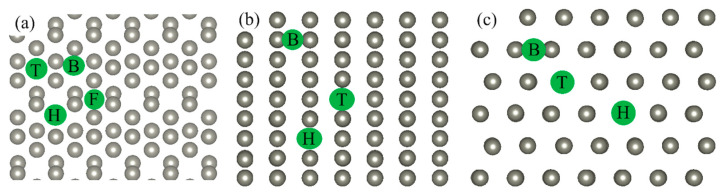
High-symmetry adsorption sites. FCC (F), hcp (H), bridge (B), and top (T) on the (**a**) Zn(111), (**b**) Zn(110), and (**c**) Zn(100) surfaces. Zn atoms and high-symmetry adsorption sites are indicated by gray balls and green balls, respectively.

**Figure 2:**
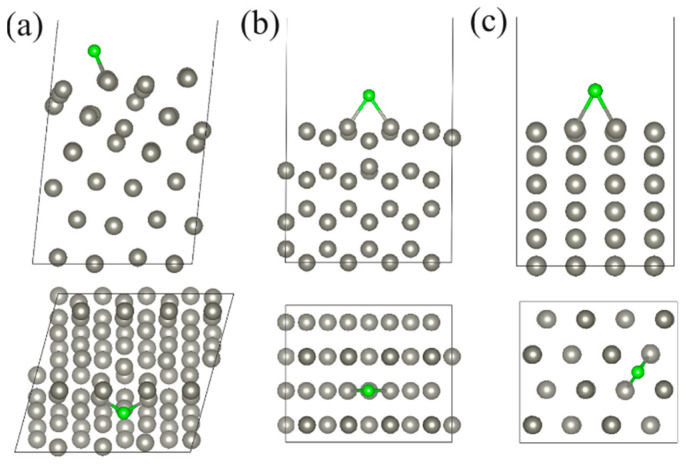
The side (the upper panel) and top (the lower panel) views of the lowest-energy configuration of the Cl atom adsorbed on the (**a**) Zn(111), (**b**) Zn(110), and (**c**) Zn(100) surfaces. Zn and Cl atoms are indicated by gray and green balls, respectively.

The interaction of corrosive atoms with Zn surfaces is considered based on various adsorption characteristics. These characteristics are calculated and collected in Table 1 and Table S1. First, the lowest energy configurations of Cl, O, and S atoms on the Zn(111), Zn(110), and Zn(100) surfaces are studied. For that, studied adsorbates are placed at the high-symmetry adsorption sites [24,25] as shown in Figure 1. According to Figure 2 and Table 1, the hcp site is the most favorable for a Cl atom on the Zn(111) and Zn(110) surfaces, while the bridge site is the preferable site for a Cl atom on the Zn(100) surface. In the case of O (Figure S1) and S (Figure S2) atoms, the lowest energy positions on the Zn(111), Zn(110), and Zn(100) surfaces are the bridge, hcp, and bridge, respectively.

Adsorption height *d* and *E*_ads_ are other representative markers of adsorption. The lower the distance from the corrosive adsorbate to the surface atom and the more negative the *E*_ads_ value is, the stronger the adsorbate binds to the surface and, consequently, the lower the resistance of the surface to the corrosion [11]. According to Table 1, the Cl atom possesses the shortest *d* = 1.54 Å and the lowest *E*_ads_ = −2.88 eV on the Zn(111) surface. For comparison, an *E*_ads_ of −4.44 eV has been previously reported for the Cl atom on the Fe(100) surface [13]; thus, Zn may be more stable than Fe in Cl-rich mediums, while both will corrode rapidly. Intercalation of the O atom to the surface with the lowest *E*_ads_ of ~−7.30 eV is observed in the case of the Zn(111) surface. The S atom adsorbed on the Zn surface has the shortest *d* of 0.81 Å and the lowest *E*_ads_ of ~−4.96 eV at the bridge site of the Zn(100) surface.

**Figure 3:**
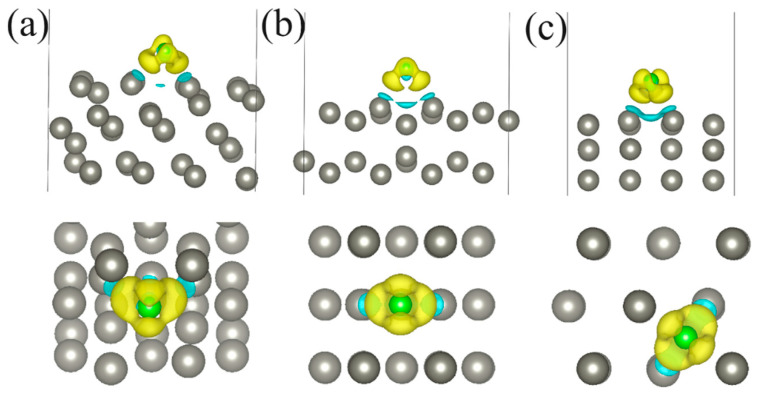
The DCD isosurface plots (0.005 Å^−3^) of the Cl atom adsorbed on (**a**) Zn(111), (**b**) Zn(110), and (**c**) Zn(100) surfaces. The yellow (blue) color represents an accumulation (depletion) of electrons.

The binding of the adsorbate atom to the surface can cause a remarkable charge redistribution on the reacting atoms. These changes can also be valuable descriptors of the corrosion process. Differential charge density (DCD) graphs and a Bader analysis are utilized to visualize and to quantify the charge transfer upon the atoms’ adsorption on Zn surfaces. Figure 3 shows the DCD plots for the Cl atom adsorbed on the Zn(111), Zn(110), and Zn(100) surfaces. The accumulation of electrons is observed in the Cl atom, while electron depletion is found on the surface Zn atoms surrounding the Cl atom. The Bader analysis (Table 1) proves the charge transfers of 0.598 e, 0.595 e, and 0.601 e, respectively, from Zn atoms on the Zn(111), Zn(110), Zn(100) surfaces to the adsorbed Cl atom. Similarly, the DCD plots in Figure S3 suggest the charge transfer occurs from the surface Zn atoms to the adsorbed O atom. The amount of the charge transferred from the Zn(111), Zn(110), and Zn(100) surfaces to the O atom is 1.231 e, 1.196 e, and 1.195 e, respectively (Table 1). According to Figure S4 and Table 1, there is a charge transferred from the surface Zn atoms to the S atom. This is confirmed by the Bader analysis, which suggests the S atom is an acceptor to the Zn(111), Zn(110), and Zn(100) surfaces with the amount of the charge transferred being 0.823 e, 0.820 e, and 0.825 e per atom, respectively.

**Figure 4:**
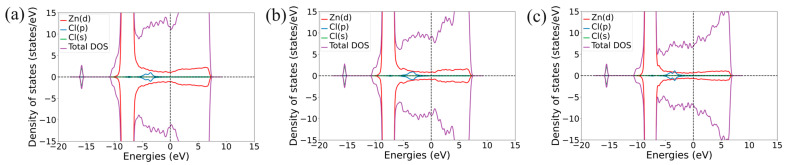
PDOS diagrams of a Cl atom adsorbed on (**a**) Zn(111), (**b**) Zn(110), and (**c**) Zn(100) surfaces.

PDOS diagrams of a Cl atom after adsorption on the Zn(111), Zn(110), and Zn(100) surfaces is shown in Figure 4a–c. Energy level-splitting of Cl-*p* orbitals produced by the spin–orbit interaction is visible in Figure 4a,c. In the case of Cl adsorbed on the Zn(111) surface (Figure 4a), the S-*p* orbital is splitting and broadening because of a strong coupling to the Zn-*d* orbital. For the Cl atom adsorbed on the Zn(110) surface (Figure 4b), there is no spin decomposition, while the Cl-*p* orbital is significantly broadened, which can be due to Cl atoms having the highest *E*_ads_ on the Zn(110) surface compared to the other surfaces considered. In the case of the Cl atom on the Zn (100) surfaces (Figure 4c), a decomposition and significant broadening of the Cl-*p* orbital is observed, indicating a strong hybridization between the Cl-*p* and the Zn-*d* orbitals. PDOS graphs for the O atom adsorbed on the Zn(111), Zn(110), and Zn(100) surfaces is shown in Figure S5a–c. A strong degeneracy of the O-*p* orbital of the O atom due to the spin–orbit interaction with the Zn-*d* orbital of the surface Zn atoms can be seen in the case of the Zn(111) (Figure S5a), Zn(110) (Figure S5b), and Zn(100) (Figure S5c) surfaces. Figure S6a–c present PDOS diagrams of the S atom adsorbed on the Zn(111), Zn(110), and Zn(100) surfaces, respectively. Energy level-splitting for the S-*p* orbital produced by the spin–orbit interaction is visible in Figure S6. In the case of S adsorbed on the Zn(111) surface (Figure S6a), the S-*p* orbital is split and broadened because of a strong coupling to the Zn-*d* orbital. For S adsorbed on the Zn(110) (Figure S6b) and Zn(100) (Figure S6c) surfaces, the S-*p* orbital is broadened, signifying coupling to the Zn-*d* orbital, while no spin decomposition is found. The PDOS plots can be useful for predicting the broadening of inner molecular orbitals of adsorbed atoms (molecules, etc.); thus, they can facilitate the prediction of selectivity and corrosion resistance of metal surfaces [26,27].

Another characteristic feature of the surface that can be changed by adsorbed atoms is the WF value [28,29,30]. The WF values for the pure and Cl-, O-, and S-adsorbed Zn(111), Zn(110), and Zn(100) surfaces are calculated and summarized in Table 2. The presence of adsorbed atoms on the Zn surface leads to a noticeable increase in the WF value. The highest WF values of 4.46 eV and 4.36 are found for the S-adsorbed Zn(111) surface and for the Cl-adsorbed Zn(110) surface, respectively. This can be explained based on the above-mentioned strong charge flows (Figure 3 and Figures S3 and S4) from the surface toward the adsorbate, which can lead to the dipole formation on the Zn surface. Such a dipole can be attributed to the WF modifications [31,32,33].

**Figure 5:**
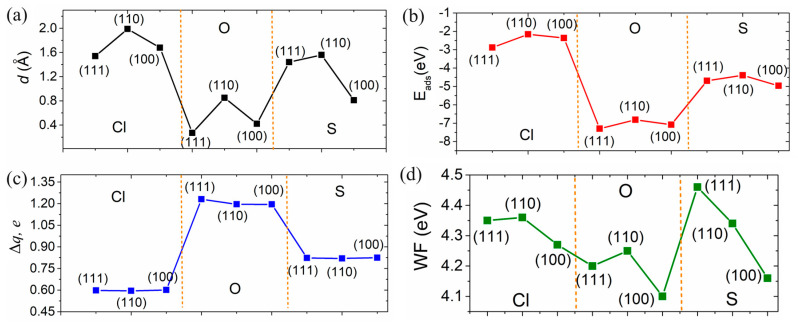
(**a**) The distance *d* between the atom and the surface, (**b**) adsorption energy *E*_a_, (**c**) Bader charge per adsorbed atom, and (**d**) WF value for the lowest-energy configurations of Cl, O, and S atoms on the Zn(111), Zn(110), and Zn(100) surfaces.

Figure 5a–d below present the descriptors collected in this work, such as adsorption height, adsorption energy, Bader charges of individual atoms, and work function values. A clear correlation between these physical characteristics can be seen. Specifically, the lower adsorption height (Figure 5a), the lower adsorption energy (Figure 5b), and the higher charge redistribution (Figure 5c) between the Cl, O, and S adsorbates and the Zn surfaces. The highest sensitivity of the Zn surfaces is attributed to the O atom, while the lowest is to the Cl atom. Notably, the change in the work function values of the Zn surfaces has an inverse relationship with the amount of charge transferred from the surface to the specific adsorbate (Figure 5d).

## 3. Conclusions

The development of an analytical model based on physical parameters characterizing the local atomic environment, such as structural parameters and electronic features can open the door to fast predictions of the adsorption energy landscape, selectivity, and chemical activity of metal surfaces toward various atomic species. However, a proper selection and comprehensive descriptions of these structural parameters and electronic features are needed. This work presents an atomic-scale consideration of corrosive Cl, O, and S atoms interacting with Zn surfaces. A strong correlation between adsorption height, adsorption energy, Bader charges of individual atoms, and work function value change is shown. Based on the descriptors presented in this work, it is concluded that a Cl-rich environment is less harmful for the Zn surface compared to an O-rich environment, and more harmful compared to a S-rich environment. Furthermore, the obtained data is collected and presented in the form of dataset. Following the collected data, one can check the sensitivity of a given Zn surface toward Cl-, O-, and S-rich corrosive media. Moreover, the presented PDOS plots can be used to evaluate the selectivity and corrosion resistance of Zn surfaces. A potential future research direction is to collect corrosion descriptors for various metal surfaces to expand the dataset for predicting their corrosion behavior.

## 4. Materials and Methods

Spin-polarized simulations were conducted based on the DFT using the Vienna Ab Initio Simulation Package (VASP) [34]. The projector augmented plane–wave method [35] was used to treat the ion–electron interactions. An energy cut-off of 540 eV was adopted for the plane–wave expansion of the electronic wave functions. The exchange correlation functional based on the general gradient approximation of Perdew–Burke–Ernzerh [36] was used in conjunction with Grimme’s DFT-D3 method [37] to accurately describe the dispersion correction of long-range van der Waals interactions between atoms and surfaces. All considered structures were fully optimized until the maximum force acting on each atom was less than 0.02 eV/Å. The change in the total energy was less than 10^4^ eV in the case of structure optimization and less than 10^8^ eV in the case of electronic self-consistent simulations. The Brillouin zone was sampled with a 4 × 4 × 1 and 8 × 8 × 1 centered *k*-mesh grid for the bulk Zn and for the surfaces of Zn, respectively. The functional and *k*-point sampling choice was based on previous work [7].

The unit cell of zinc was optimized using variable–cell relaxation, where all degrees of freedom, such as volume, shape, and internal atomic positions were allowed to relax for structural optimization. The calculated lattice parameters of zinc of *a* = *b* = 2.64 and *c* = 4.71 Å was found to be in good agreement with the literature. The surfaces of Zn were supposed to have different reactivities. For instance, the 100 surface has the lowest surface energy, thus it may be more favorable for adsorption, while the 111 and 110 surfaces possess a much larger surface energy, thus they may be less favorable for adsorption [7,38]. Therefore, these 111, 110, and 100 surfaces were considered. The size of the created Zn slabs was selected to avoid self-interaction of the replicated cells. A vacuum space of 20 Å was used in the out-of-plane direction relative to the surface plane.

The strength of adsorption of an atom to a Zn slab surface is measured based on the *E*_ads_, which is determined as follows:

*E*_ads_ = *E*_slab+a_ − *E*_a_ − *E*_slab_, (1)

where *E*_slab+a_ is the energy of the atom adsorbed on the slab surface, *E*_a_ is the energy of the pure atom, and *E*_slab_ is the energy of the pure slab surface.

The DCD Δ*ρ*(r) is determined as follows:

Δ*ρ*(r)= *ρ_E_*_slab+a_(r) − *ρ*_a_(r) − *ρ_E_*_slab_(r), (2)

where, *ρ_E_*_slab+a_(r), *ρ*_a_(r), and *ρ_E_*_slab_(r) are the charge densities of the atom-adsorbed slab surface, the pure atom, and the pure slab surface.

The WF is determined as follows:

WF = *E*_vac_ − *E*_Fermi_ (3)

where *E*_vac_ is the energy level of a stationary electron in the vacuum and *E*_Fermi_ corresponds to the Fermi level of the system.

The amount of charge transferred between the atom and the slab surface due to adsorption was quantitatively calculated using Bader charge analysis [39]. Structural and DCD analysis and plotting were conducted using VESTA 3 programs [40], while the PDOS analysis and plotting were conducted utilizing an open-source programming language (Python) [41].

## Figures and Tables

**Table 1:** Results for the lowest-energy configurations of Cl, O, and S atoms on the Zn(111), Zn(110), and Zn(100) surfaces. The distance *d* between the atom and the surface, adsorption energy *E*_a_, and the amount of charge transfer ∆*q* to/from the atom on the surface. A positive (negative) Δ*q* indicates a loss (gain) of electrons.

Structure	Position	*d*, Å	*E*_a_, eV	Doping Nature	Δ*q*, e
Zn(111) + Cl	hcp	1.54	−2.88	acceptor	0.598
Zn(110) + Cl	hcp	1.99	−2.16	acceptor	0.595
Zn(100) + Cl	bridge	1.68	−2.38	acceptor	0.601
Zn(111) + O	bridge	0.27	−7.30	acceptor	1.231
Zn(110) + O	hcp	0.85	−6.81	acceptor	1.196
Zn(100) + O	bridge	0.42	−7.08	acceptor	1.195
Zn(111) + S	bridge	1.44	−4.69	acceptor	0.823
Zn(110) + S	hcp	1.56	−4.39	acceptor	0.820
Zn(100) + S	bridge	0.81	−4.96	acceptor	0.825

**Table 2:** WF (eV) values for the cases of the lowest-energy configurations of Cl, O, and S atoms on the Zn(111), Zn(110), and Zn(100) surfaces.

Structure	Pure	Cl	O	S
Zn(111)	4.18	4.35	4.20	4.46
Zn(110)	4.15	4.36	4.25	4.34
Zn(100)	4.03	4.27	4.10	4.16

## Data Availability

The raw data supporting the conclusions of this article will be made available by the authors upon request.

## Supplementary Materials

The following supporting information can be downloaded at: https://www.mdpi.com/article/10.3390/molecules29163790/s1, Table S1: The distance *d* between the atom and the surface, adsorption energy *E*_a_, and the amount of charge transfer ∆*q* to/from the atom on the surface. A positive (negative) Δ*q* indicates a loss (gain) of electrons.; Figure S1: The side (the upper panel) and top (the lower panel) views of the lowest-energy configuration of the O atom adsorbed on (a) Zn(111), (b) Zn(110), and (c) Zn(100) surfaces.; Figure S2: The side (the upper panel) and top (the lower panel) views of the lowest-energy configuration of the S atom adsorbed on (a) Zn(111), (b) Zn(110), and (c) Zn(100) surfaces.; Figure S3: The DCD isosurface plots (0.005 Å^−3^) of the O atom adsorbed on (a) Zn(111), (b) Zn(110), and (c) Zn(100) surfaces. The green (blue) color represents an accumulation (depletion) of electrons.; Figure S4: The DCD isosurface plots (0.005 Å^−3^) of the S atom adsorbed on (a) Zn(111), (b) Zn(110), and (c) Zn(100) surfaces. The green (blue) color represents an accumulation (depletion) of electrons.; Figure S5: PDOS diagrams of O atom (a) before interaction and adsorbed on (b) Zn(111), (c) Zn(110), and (d) Zn(100) surfaces.; Figure S6: PDOS diagrams of (a) S atom before interaction and S atom adsorbed on (b) Zn(111), (c) Zn(110), and (d) Zn(100) surfaces.
