# Supplementary material for: Density Functional Theory-Based Indicators to Estimate the Corrosion Potentials of Zinc Alloys in Chlorine-, Oxidizing-, and Sulfur-Harsh Environments
Source: Molecules. 2024 Aug 10;29(16):3790. doi: 10.3390/molecules29163790 (PMC11357478; doi:10.3390/molecules29163790)
Supplement: Supplementary file 1 [file molecules-29-03790-s001.zip › molecules-3157618-supplementary.pdf]

Supplementary Materials

for

Density functional theory based indicators to estimate the corrosion potentials of zinc alloys in chlorine-, oxidizing- and sulfur-harsh environments

Azamat Mukhametov[1,^], Insaf Samikov[1^], Elena A. Korznikova[1,2], Andrey A. Kistanov[1],*

[1]The Laboratory of Metals and Alloys Under Extreme Impacts, Ufa University of Science and Technology, Ufa 450076, Russia;

[2]Polytechnic Institute (Branch) in Mirny, North-Eastern Federal University, Mirny 678170, Sakha Republic, Russia.

***** Correspondence: andrei.kistanov.ufa@gmail.com;

^ These authors contributed equally.

Table S1. The distance *d* between the atom and the surface, adsorption energy E_a, and the amount of charge transfer Δq to/from the atom on the surface. A positive (negative) Δq indicates a loss (gain) of electrons.

Structure	Position	d, Å	E_a, eV	Doping nature	Δq, *e*
Zn(111)+Cl	top	1.55	-2.78		
Zn(111)+Cl	bridge	1.46	-2.81		
Zn(111)+Cl	**hcp**	**1.54**	**-2.88**	**acceptor**	**0.598**
Zn(111)+Cl	fcc	1.55	-2.86		
Zn(110)+Cl	top	2.22	-1.81		
Zn(110)+Cl	**hcp**	**1.99**	**-2.16**	**acceptor**	**0.595**
Zn(110)+Cl	bridge	1.99	-2.15		
Zn(100)+Cl	top	2.19	-2.08		
Zn(100)+Cl	hcp	1.98	-2.06		
Zn(100)+Cl	**bridge**	**1.68**	**-2.38**	**acceptor**	**0.601**
Zn(111)+O	top	0.91	-6.95		
Zn(111)+O	fcc	0.90	-6.96		
Zn(111)+O	hcp	0.83	-7.12		
Zn(111)+O	**bridge**	**0.27**	**-7.30**	**acceptor**	**1.231**
Zn(110)+O	top	0.99	-6.32		
Zn(110)+O	**hcp**	**0.85**	**-6.81**	**acceptor**	**1.196**
Zn(110)+O	bridge	0.84	-6.80		
Zn(100)+O	top	0.42	-7.08		
Zn(100)+O	hcp	0.46	-6.70		
Zn(100)+O	**bridge**	**0.42**	**-7.08**	**acceptor**	**1.195**
Zn(111)+S	top	1.50	-4.68		
Zn(111)+S	**bridge**	**1.44**	**-4.69**	**acceptor**	**0.823**
Zn(111)+S	fcc	1.50	-4.67		
Zn(111)+S	hcp	1.47	-4.68		
Zn(110)+S	top	1.63	-4.10		
Zn(110)+S	bridge	1.40	-4.18		
Zn(110)+S	**hcp**	**1.56**	**-4.39**	**acceptor**	**0.820**
Zn(100)+S	top	1.04	-4.49		
Zn(100)+S	hcp	0.69	-4.92		
Zn(100)+S	**bridge**	**0.81**	**-4.96**	**acceptor**	**0.825**

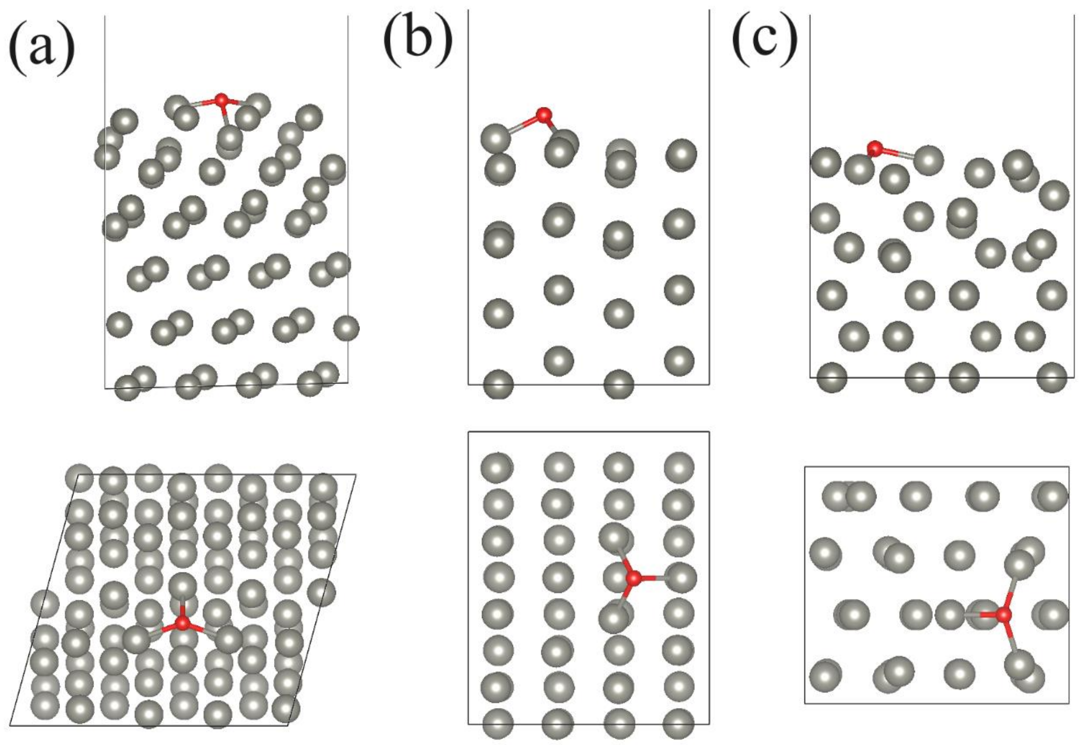

Figure S1. The side (the upper panel) and top (the lower panel) views of the lowest-energy configuration of the O atom adsorbed on (a) Zn(111), (b) Zn(110), and (c) Zn(100) surfaces.

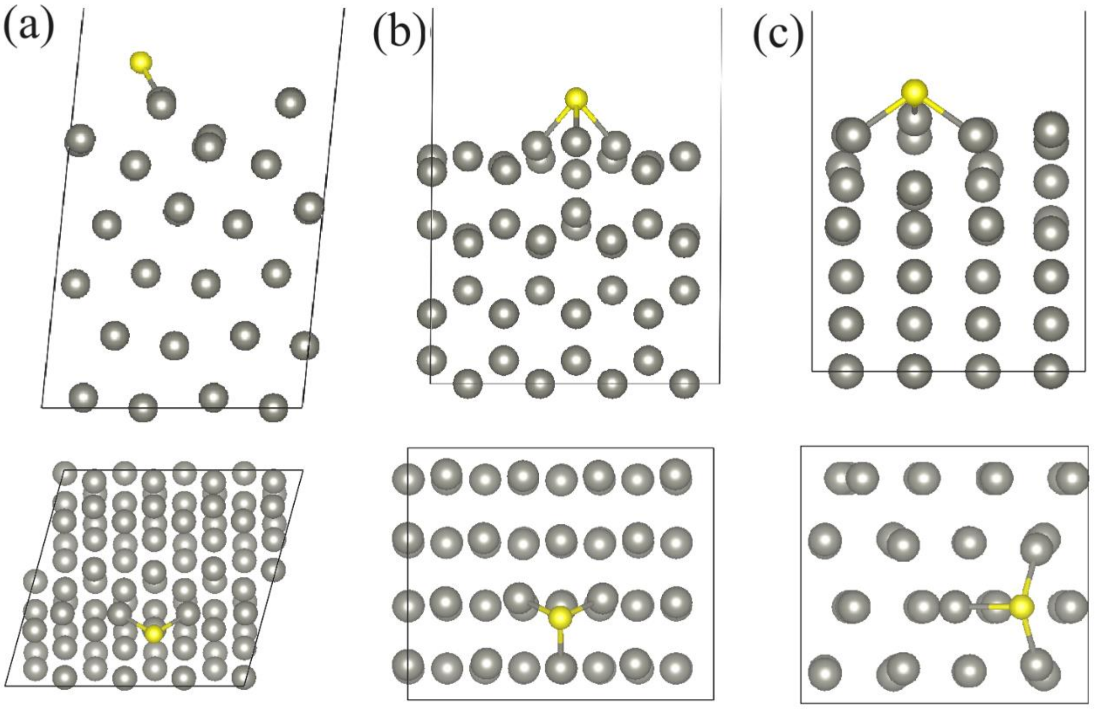

Figure S2. The side (the upper panel) and top (the lower panel) views of the lowest-energy configuration of the S atom adsorbed on (a) Zn(111), (b) Zn(110), and (c) Zn(100) surfaces.

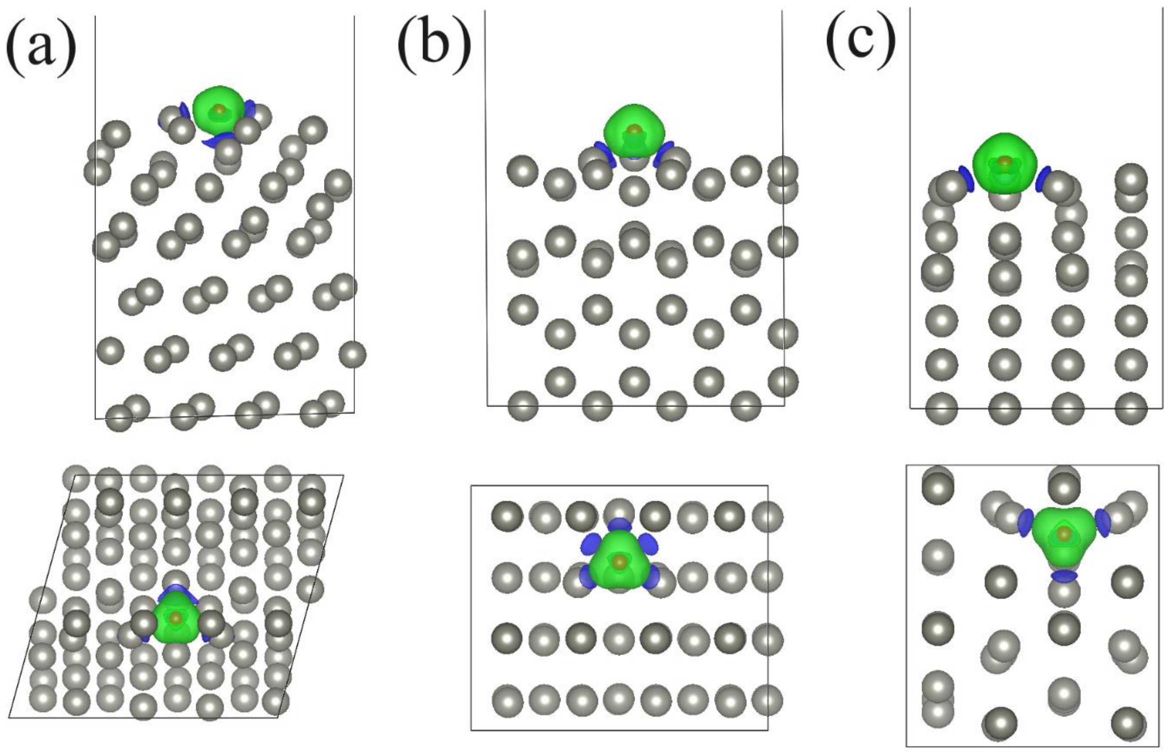

Figure S3. The DCD isosurface plots (0.005 Å^{-3}) of the O atom adsorbed on (a) Zn(111), (b) Zn(110), and (c) Zn(100) surfaces.. The green (blue) color represents an accumulation (depletion) of electrons.

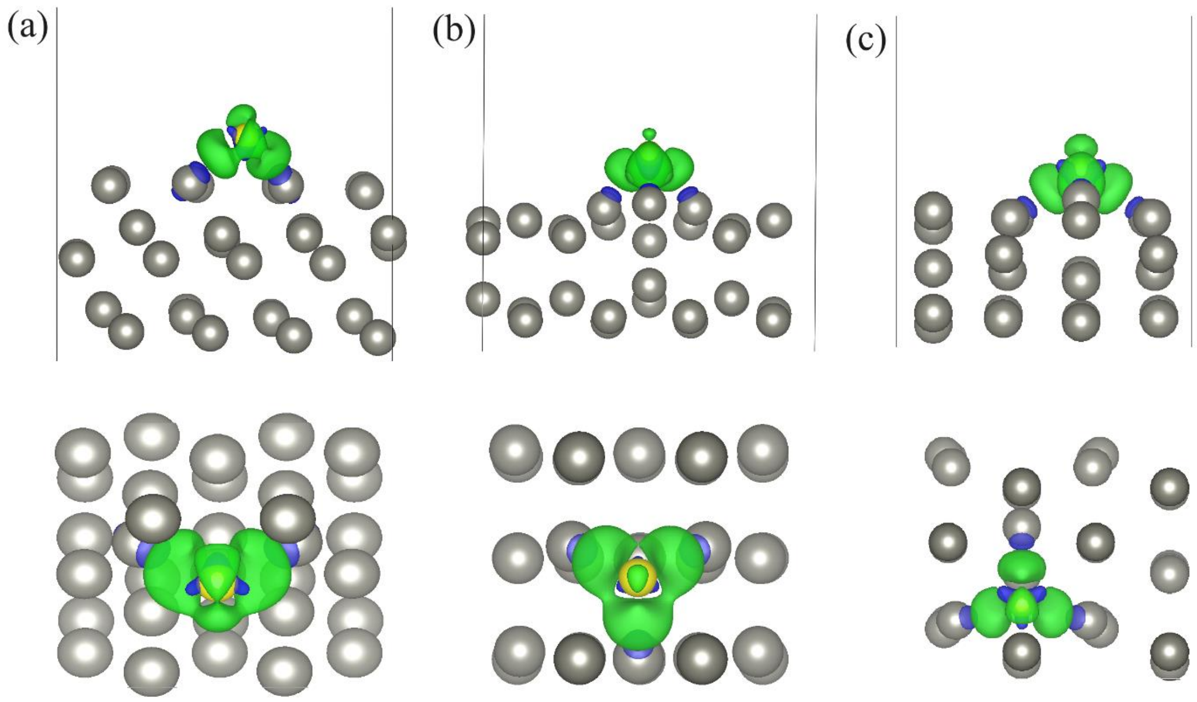

Figure S4. The DCD isosurface plots (0.005 Å^{-3}) of the S atom adsorbed on (a) Zn(111), (b) Zn(110), and (c) Zn(100) surfaces.. The green (blue) color represents an accumulation (depletion) of electrons.

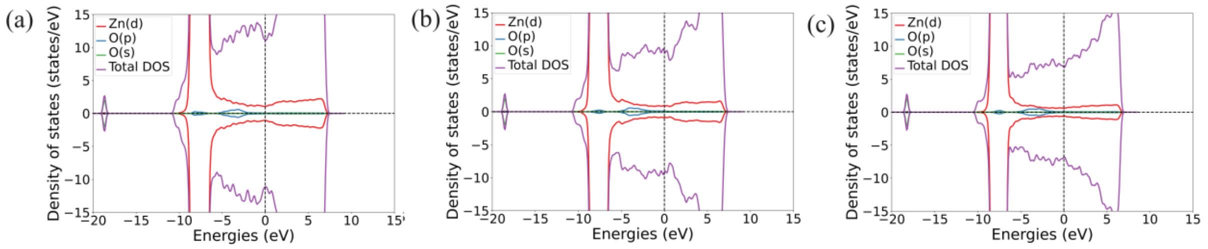

Figure S5. PDOS diagrams of O atom (a) before interaction and adsorbed on (b) Zn(111), (c) Zn(110), and (d) Zn(100) surfaces.

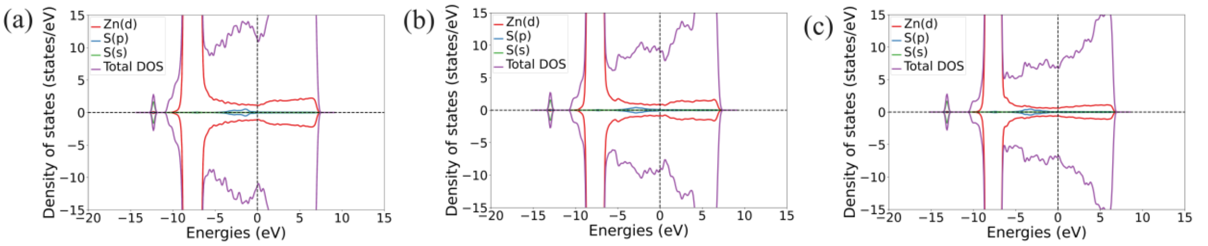

Figure S6. PDOS diagrams of (a) S atom before interaction and S atom adsorbed on (b) Zn(111), (c) Zn(110), and (d) Zn(100) surfaces.
